# The Effects of Extrinsic and Intrinsic Factors on Neurogenesis

**DOI:** 10.3390/cells12091285

**Published:** 2023-04-29

**Authors:** Mei Jiang, Se Eun Jang, Li Zeng

**Affiliations:** 1 Department of Human Anatomy, Dongguan Key Laboratory of Stem Cell and Regenerative Tissue Engineering, Dongguan Campus, Guangdong Medical University, Dongguan 523808, China; 2 Neural Stem Cell Research Lab, Research Department, National Neuroscience Institute, Singapore 308433, Singapore; 3 Neuroscience and Behavioral Disorders Program, DUKE-NUS Graduate Medical School, Singapore 169857, Singapore; 4 Lee Kong Chian School of Medicine, Nanyang Technology University, Novena Campus, 11 Mandalay Road, Singapore 308232, Singapore

**Keywords:** neural stem cell, neurogenesis, stem cell regeneration, neurological impairments, SARS-CoV-2

## Abstract

In the mammalian brain, neurogenesis is maintained throughout adulthood primarily in two typical niches, the subgranular zone (SGZ) of the dentate gyrus and the subventricular zone (SVZ) of the lateral ventricles and in other nonclassic neurogenic areas (e.g., the amygdala and striatum). During prenatal and early postnatal development, neural stem cells (NSCs) differentiate into neurons and migrate to appropriate areas such as the olfactory bulb where they integrate into existing neural networks; these phenomena constitute the multistep process of neurogenesis. Alterations in any of these processes impair neurogenesis and may even lead to brain dysfunction, including cognitive impairment and neurodegeneration. Here, we first summarize the main properties of mammalian neurogenic niches to describe the cellular and molecular mechanisms of neurogenesis. Accumulating evidence indicates that neurogenesis plays an integral role in neuronal plasticity in the brain and cognition in the postnatal period. Given that neurogenesis can be highly modulated by a number of extrinsic and intrinsic factors, we discuss the impact of extrinsic (e.g., alcohol) and intrinsic (e.g., hormones) modulators on neurogenesis. Additionally, we provide an overview of the contribution of severe acute respiratory syndrome coronavirus 2 (SARS-CoV-2) infection to persistent neurological sequelae such as neurodegeneration, neurogenic defects and accelerated neuronal cell death. Together, our review provides a link between extrinsic/intrinsic factors and neurogenesis and explains the possible mechanisms of abnormal neurogenesis underlying neurological disorders.

## 1. Introduction

The central nervous system (CNS) is the most important component of the human nervous system and receives and integrates afferent inputs from all over the body, playing a crucial role in brain functions such as learning, memory and emotion [1]. The CNS contains approximately 86.1 billion neurons and an even greater number of glial cells [2,3]. In the second half of the 20th century, it was accepted in the field of neuroscience that the adult mammalian brain could not generate new neurons because the structure of the brain was fixed. However, this doctrine was overturned following the seminal discovery of Joseph Altman and colleagues that adult neurogenesis occurs in several mammalian species [4,5]. Following several decades of exploration by researchers using different techniques to label new neurons, studies have found that the brains of adult mammals and humans contain new neurons, which has been confirmed in vitro and in vivo [4,6,7]. We now understand that neurogenesis can occur in the embryonic and adult brains in a wide range of vertebrates, from fish to primates [8,9,10], and this provides a direction to explore neuronal plasticity and cognition in the CNS.

It has been reported that symptoms such as depression, anxiety, cognitive, or olfactory dysfunction are observed in the early stages of neurodegenerative disorders such as Parkinson’s disease (PD), Alzheimer’s disease (AD) and Huntington’s disease (HD) prior to disease-specific patterns of neurite degeneration and neuronal loss [11,12,13]. This demonstrates that these diseases are linked to the primary regions responsible for adult neurogenesis, such as olfactory areas and the hippocampus. Specifically, impaired adult neurogenesis may be associated with the early symptoms of neurodegenerative diseases in some patients. Therefore, the fact that adult neurogenesis is impaired in neurodegenerative diseases indicates that, in such diseases, cell self-renewal in the adult brain and the putative function of newborn neurons are compromised or lost, in addition to the number of existing neurons being reduced. Interestingly, neurogenesis can be affected by intrinsic factors if molecular signaling occurs intracellularly or by extrinsic factors if signaling pathways are mediated by components of the extracellular microenvironment [14].

This review provides a detailed discussion of the effects of extrinsic and intrinsic factors on neurogenesis, as well as the molecular mechanism underlying neurogenesis, which may provide better insight into the mechanisms of neurodegenerative disorders and new directions for their treatment.

## 2. Neurogenesis

### 2.1. Locations and Origins of Neural Stem Cells

In 1992, Reynolds et al. described a cell population isolated from the striatum of the adult mouse brain that could divide and proliferate continuously in vitro and had the potential to differentiate into a variety of cell types; this discovery of NSCs challenged the traditional theory that adult mammalian nerve cells cannot regenerate [15]. Subsequently, McKay and colleagues provided additional evidence for the function of NSCs in 1997, demonstrating that NSCs are pluripotent stem cells in the nervous system and have the potential to differentiate into neurons and glia [16]. During embryonic development, there are two crucial NSC niches, the ventricular zone (VZ) and subventricular zone (SVZ), where cortical neurons and glial cells originate. Neural stem cells located in the VZ of the neural tube participate in forming the CNS by differentiating into required cell types. There are two regions in the adult mammalian CNS that contain NSCs: the SVZ of the lateral ventricle and the subgranular zone (SGZ) of the hippocampal dentate gyrus (DG) [17]. Using the immunofluorescence labeling of SVZ stem cells in vivo, proliferating cells can be clearly observed migrating to the olfactory bulb (OB) ventricle trajectory [18]. Subgranular zone stem cells form granule cells and gradually establish synaptic connections in the hippocampal DG. Neural stem cells play an important role in maintaining physiological function and forming the developing brain in the prenatal and postnatal periods and in the regenerating of nerve tissue in injured regions of the adult brain [19,20].

Neural stem cells are undifferentiated primitive cells that do not express mature cellular antigens, have low immunogenicity and are not recognized by the immune system. In addition, NSCs can coexist well with host neural tissue and survive in the host for a long time [21]. Therefore, NSCs are able to survive for a longer period of time when transplanted. Additionally, a large number of NSCs exist in the nervous system of mammals, including humans, during development and adulthood. Neural stem cells can be obtained from a variety of sources: (1) neural tissue [22]; (2) embryonic stem cells, embryonic germ cells and other cells [23]; (3) bone marrow stromal stem cells (BMSCs) (via epidermal growth factor (EGF) or brain-derived neurotrophic factor (BDNF) application) [24], multipotent adult progenitor cells (MAPCs) (when copurified with mesenchymal stem cells) and umbilical cord blood cells (can be induced to differentiate into multipotential stem cells by nerve growth factor (NGF) and retinoic acid (RA)) [25] from the blood system; (4) the immortalized cell lines C17.2 (generated by the retrovirus-mediated transduction of avian Myc into immortalized neonatal mouse cerebellar precursor cells) [26]; (5)the Maudsley hippocampal cell line, clone 36 (MHP36) (an immortalized murine stem cell line generated by the transduction of the temperature-sensitive oncogene tsA58 into the hippocampal proliferative zone of mice on embryonic day 14; temperature-sensitive NSCs that were implanted into the brain at different temperatures to generate cells with different phenotypes) [27] and NTerra-2 (NT2) (obtained from human testicular germ cell tumors and show the characteristics of NSCs induced by RA) [27]; and (6) the generation of embryonic stem cells by nuclear transfer from adult somatic cells [28]. The source of NSCs may be selected depending on the mechanism by which NSCs might improve function.

### 2.2. Developmental Neurogenesis

Neurogenesis is a critical process throughout life and involves the generation of new functional neurons from NSC NPC and neuroblast proliferation, the differentiation, maturation and maintenance of neurons and the migration and integration of new neurons into existing brain circuits [29]. These steps are interdependent and partially overlap.

**Figure 1:**
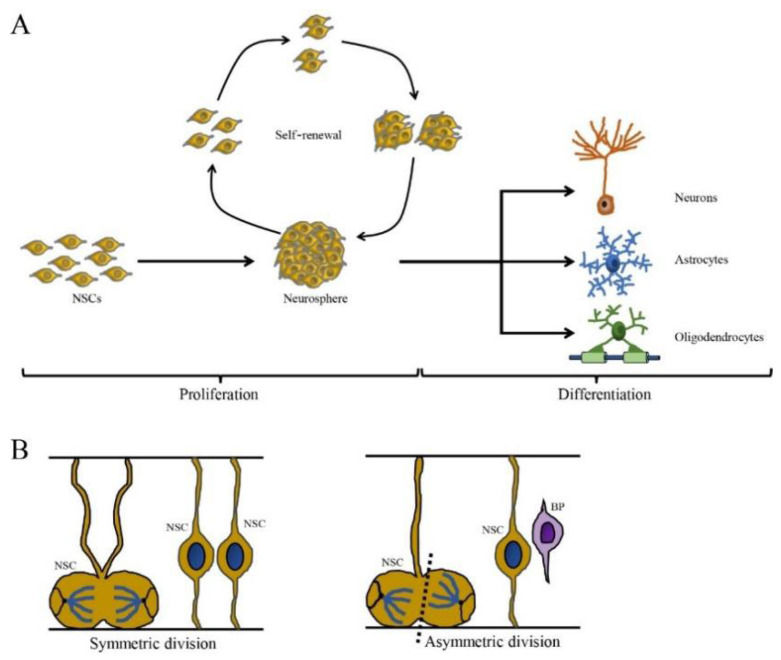
The self-renewing capacity and cell division pattern of NSCs. (**A**) The NSCs are self-renewing and multipotent cells with the ability to produce neurons, astrocytes and oligodendrocytes. (**B**) The cell division of NSCs is divided in two ways: symmetric division (generated two daughter NSCs) and asymmetric division (generate one NSC and one basal progenitor (BP) cell) (cited by Ribeiro, 2021).

Neural stem cells undergo proliferation and self-renewal by symmetric and asymmetric cell division to maintain the NSC pool [17]. Symmetrical division produces two progeny stem cells, while asymmetric division generates one progeny stem cell and one differentiated progeny cell that eventually differentiates into a terminal cell (Figure 1). Neural stem cells proliferate as nonadherent spherical structures called neurospheres, which are composed of clonally derived precursors. Neural stem cells unceasingly proliferate and then give rise to many offspring neurospheres with the same phenotype, thus showing self-renewal capacity [30,31]. However, the number of NSCs can be decreased by multiple nonexclusive mechanisms including the inhibition of proliferation, increased cell death and/or greater commitment to other cell lineages. For example, viral infection blocks specific stages of the cell cycle and induces DNA damage, which may inhibit proliferation and lead to the depletion of the NSC pool, which is associated with a range of neurological outcomes including microcephaly, memory deficits and cognitive decline [32,33,34].

Neural stem cells also have the capacity to differentiate into neurons, oligodendrocytes and astrocytes, which are closely linked to factors in the local microenvironment such as growth factors. During brain development, NSCs differentiate in a temporal manner, with neurogenesis occurring first, followed by gliogenesis. In rodents, neurogenesis begins in the early embryonic stages (E10~E11) and continues until birth, although dendritic and axonal maturation continue postnatally. Gliogenesis begins in the late embryonic stages and peaks at birth [35,36]. In humans, both neurogenesis and gliogenesis occur primarily during gestation, but development continues even after birth [37]. The switch in neurogenesis to gliogenesis during development is strictly modulated by basic helix-loop-helix (bHLH) transcription factors and signaling pathways such as bone morphogenetic proteins (BMPs), Notch and the Janus kinase/signal transducer and activator of the transcription (JAK-STAT) pathway [38,39]. Differentiation can be disrupted by extrinsic and intrinsic factors in multiple ways, including the inhibition of a single lineage, a shift to alternative lineages, or the broad inhibition of differentiation into multiple cell types. Aberrant differentiation is associated with the impaired development of the brain and neurological diseases, resulting in neurodevelopmental, neurodegenerative and neuropsychiatric disorders [40,41]. For example, autism spectrum disorder (ASD) occurs when the neuronal density in the prefrontal cortex is increased during childhood due to accelerated neuronal differentiation [42,43].

In addition, resident NSCs in the SVZ undergo asymmetric neurogenic cell division to generate daughter cells that differentiate into restricted neuroblasts prior to migration to the OB via the rostral migratory stream (RMS) [44]. Furthermore, NSCs located in the SGZ produce daughter cells that migrate a relatively short distance to the DG. After reaching their final destination, restricted neuroblasts differentiate into immature neurons and further develop the morphological and electrophysiological characteristics of mature neurons [45]. The stage of NSC maturation is an important variable that determines susceptibility to infection and impacts differentiation. Undifferentiated stem cells are often more susceptible to viral infections and subsequent cell death [46]. However, it has been reported that newborn neurons are also produced in other structures of the brain and we will further discuss this topic in this review.

### 2.3. Niches of Adult Neurogenesis

Just before birth, RGCs produce NSCs, which serve as a pool for adult neurogenesis and developmental processes [47,48]. In the adult mammalian brain, there are two primary neurogenic niches, the SVZ in the lateral ventricle and the SGZ of the hippocampal DG. In these niches, NSCs generate NPCs, which are more capable of self-renewal, or transit-amplifying progenitors, which rapidly divide to form migrating neuroblasts and then differentiate into neurons that integrate into preexisting circuits. Adult neurogenesis occurs not only under physiological conditions but also upon brain injury to regulate endogenous repair mechanisms [49]. Here, we provide a comprehensive overview of the process of adult neurogenesis in different brain regions.

#### 2.3.1. Subventricular Zone (SVZ)

**Figure 2:**
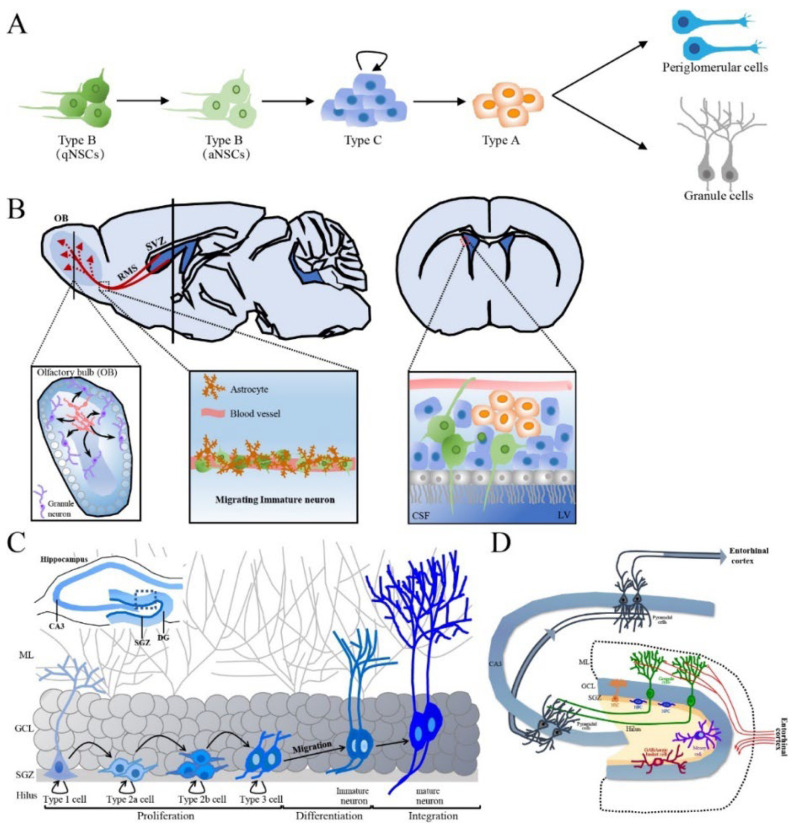
The production process of newborn neurons in adult SVZ and SGZ and the integration of newly born neurons in hippocampus. (**A**) Schematic representation of adult SVZ neurogenesis in five developmental stages. (**B**) The sagittal section and cross section of the brain, the synaptic integration and maturation of granule cell and periglomerular in the OB, the composition of RMS as well as the connection between NSCs of SVZ and CSF of LV are shown. Green cells are type B cells, blue cells are type C cells and orange cells are type A cells. (**C**) Schematic representation of adult hippocampal neurogenesis in the hippocampal DG. (**D**) The structure and circuits of the hippocampus. Schematic representation of a transversal slice of the hippocampus depicting the DG, CA3 and SGZ. SVZ: subventricular zone, NSC: neural stem cell, qNSCs: quiescent NSCs, aNSCs: activated NSCs, RMS: rostral migratory stream, CSF: cerebrospinal fluid, LV: lateral ventricle, DG: dentate gyrus, CA: cornus ammonis, SGZ: subgranular zone, ML: molecular layer, GCL: granular cell layer, OB: olfactory bulb.

The SVZ is situated along the ependymal cell layer of the lateral walls of the lateral ventricles and contains a subpopulation of resident NSCs in the resting state (also called type B cells) that have radial-glial-like characteristics and form the vasculature, ending with blood vessels in the SVZ [50]. In the SVZ, activated type B cells undergo slow asymmetric division to produce intermediate progenitors (IPCs) or transit-amplifying progenitor cells, known as type C cells. In turn, type C cells mostly generate neuronal progenitor cells (neuroblasts, type A cells) and differentiate into periglomerular cells and granule cells [51] (Figure 2A). Type A cells undergo tangential migration over a long distance along the RMS by forming a chain composed of 30~40 cells that lie close together and are encased in a meshwork of specialized GFAP-positive astrocytes, which jointly create a tubular structure from caudal to rostral longitudinally linking the SVZ to the OB [52]. Externally, astrocytes surround the chains of neuroblasts and form glial tubes, which help to separate the chains of neuroblasts from the nearby brain parenchyma and maintain long-distance migration (Figure 2B). These specialized astrocytes can overexpress nonsoluble factors, such as cell adhesion molecules and extracellular matrix, and promote neuroblast migration in a dependent manner [53]. In the OB, neuroblasts detach from the rostrally migrating chain in the RMS. Then, the neuroblasts begin to radially migrate to their final locations and differentiate into olfactory granule cells (GCs) and periglomerular cells, which integrate into the neuronal circuitry in the OB [54]. Another crucial aspect of adult neurogenesis is the regulation of newborn cell survival. Each day, thousands of newborn cells are generated in the SVZ, and some of these must be eliminated to ensure that a stable and sufficient number of new OB neurons is available. This process is controlled by the regulation of programmed cell death and is important for maintaining well-organized migration along the RMS [55]. Neuroblasts migrate to the OB under healthy conditions, but neuroblast migration can also be invoked as needed. For example, after brain injury neuroblasts migrate individually, but a similar chain of neuroblasts migrating toward injured areas forms [56]. In the OB, continuous cell turnover occurs via neurogenesis, with newly born granule cells thought to replace older ones, while cell death occurs throughout the whole SVZ~OB pathway.

In addition, type B cells maintain direct contact with the cerebrospinal fluid (CSF) in the lateral ventricles by extending their apical end into the ependymal layer (Figure 2B). The adult choroid plexus in the ventricle lumen produces various cytokines and trophic factors and secretes them into the CSF; the choroid plexus is involved in regulating the self-renewal ability, proliferation and differentiation of SVZ NSCs and NPCs. Type B cells also contact blood vessels through the extension of basal processes [57].

#### 2.3.2. Hippocampus

The hippocampus is the primary brain region responsible for memory consolidation, in which the highly orchestrated neuronal network between the entorhinal cortex, DG and cornu ammonis is essential [58]. The other major NSC pool in the adult mammalian brain is the SGZ of the hippocampal DG where adult-born granule cells are generated from neural progenitor cells, mature and integrate into the preexisting network. The V-shaped DG consists of three layers, i.e., from inside to outside, the hilus or the polymorphic cell layer, which possesses a multitude of cell types such as mossy cells; the granule cell layer (GCL), which is mainly composed of densely packed granule cells with a thickness of 6~8 neurons and encloses the first layer and the molecular layer (MCL), which is mainly occupied by the dendrites of the dentate granule cells and the fibers of the perforant path from the entorhinal cortex [59]. The SGZ is a thin band of cells located between the inner granule cell layer and the hilus of the DG that contains NSCs and NPCs as well as the bodies of parvalbumin-expressing interneurons such as GABAergic basket cells [60].

Local NSCs in the SGZ, also called type 1 cells, have the molecular characteristics of astrocytes and the morphology of radial glia-like (RGL) cells, resembling embryonic neural progenitors, and have self-renewal and multipotential capacities [61,62]. Type 1 cells are usually quiescent NSCs (qNSCs) and rarely divide but can be activated by internal and external stimuli. Activated NSCs (aNSCs) produce intermediate progenitor cells or an amplifying population of non-RGL progenitor cells (type 2 cells) via asymmetrical division. However, type 2 cells exhibit higher proliferative activity than type 1 cells and thus expand the SGZ pool of precursor cells [63]; type 2 cells have a simpler morphology than type 1 cells but have horizontally oriented short processes rather a radial morphology [64]. These type 2 cells, which express Sox2 and Nestin but not GFAP, can differentiate into two distinct subsets, i.e., type 2a and 2b cells, which can be distinguished by the expression of different proteins. Resembling type 1 cells, type 2a cells express glial markers and subsequently give rise to type 2b cells expressing neuronal lineage markers such as doublecortin (DCX, an immature neuronal marker) and the polysialylated form of the neural cell adhesion molecule (PSA-NCAM) and lacking Nestin and glial markers [64,65]. Type 3 cells are produced from type 2b cells mainly via symmetrical division but also asymmetrical division. Type 3 cells display a neuronal lineage and little proliferative activity and have either tangential or radial processes [66]. These cells migrate radially short distances to the GCL, where they exit the cell cycle and develop to form granule neurons. Therefore, the process of adult hippocampal neurogenesis can be separated into five stages: (1) the activation of qNSCs (type 1 cells) in the SGZ; (2) the amplification of nonradial NSCs and intermediate progenitors (type 2 cells); (3) the generation of neuroblasts (type 2b cells) through lineage selection; (4) the migration of immature neurons (early type 3 cells) and (5) the integration and maturation of adult-born dentate granule cells (late type 3 cells) (Figure 2C).

During the process of neurogenesis, a subset of newborn cells is generated in the SGZ each day, while most cells die by a two-step process. A large portion of cell death occurs during the early developmental period (24~48 h postmitosis) via apoptosis. Surviving cells in this stage migrate to the GCL and continue to mature [66]. Cell death also occurs during network integration, and surviving neurons are integrated into existing neuronal circuits. Surviving granule cells extend their dendrites toward the MCL of the DG to establish synaptic inputs from the entorhinal cortex and project their axons to the CA3 region of the hippocampus through the mossy fiber pathway in the hilus, which is integrated into the existing network [67,68]. When the somata of developing neurons in the SGZ reach the GCL and their dendrites extend to the MCL, the first synapses are established [60]. During synaptogenesis, the number of dendritic spines increases with dendritic length. Furthermore, the dendrites on the axons of newly generated neurons that project toward the CA3 region extend toward the MCL. Eventually, the integration of new neurons into the preexisting network can change its function [69] (Figure 2D). Although adult hippocampal neurogenesis is important for hippocampus-dependent functions, including plasticity, cognition, learning, spatial memory, pattern separation and some emotional behaviors, such as depression and anxiety [70,71], the mechanisms underlying adult hippocampal neurogenesis in the healthy aged brain have yet to be fully explored.

#### 2.3.3. Hypothalamus

The hypothalamus is known for its role in regulating autonomic function and body homeostasis and has recently been identified as a third neurogenic niche. Pioneering studies demonstrating the neurogenic capacity of the hypothalamus were first conducted to aid the growth and expansion of differentiated postnatal neurons, which were originally thought to be challenging due to the terminally differentiated state of the neurons [72]. Neurons of the adult rat hypothalamus and brainstem were cultured and stained with antibodies against the immature neuronal postmitotic marker α−internexin along with neuronal markers such as βIII-Tubulin, MAP2, Tau and tetanus toxin to confirm the presence of neurons. In addition, these cells have been shown to have early neuron-like characteristics through electrophysiology studies [72]. Next, hypothalamic progenitor cells were isolated so that neurogenically silent cells in vivo could be propagated in vitro to generate a wide spectrum of neurons destined to become neuropeptide-expressing neurons [73] and cell proliferation was increased in vivo when obese mice were stimulated with ciliary neurotrophic factor (CNTF) [74]. Both β2-tanycytes and α2−tanycytes located in the median eminence (MEm), also named the hypothalamic proliferative zone (HPZ), were identified as highly neurogenic cells within the hypothalamic region [75,76,77]. Interestingly, in the same study the authors observed a time-specific increase in neurogenesis in mice fed a high-fat diet compared to mice fed normal chow on postnatal Day 75 but not on postnatal Day 35 or 45. Despite mounting evidence of the ability of hypothalamic cells to form neurospheres [78,79] and the ability of cell proliferation and neurogenesis to be induced by stimulation [74,80,81], little is known about the HPZ and its physiological role in hypothalamic neurogenesis [82,83]. Thus, more studies are required to link findings from animal studies to human physiology.

#### 2.3.4. Striatum

The striatum plays a key regulatory role in the dopamine pathway, and the dysfunction of this system can lead to cognitive and psychiatric diseases such as schizophrenia and Parkinson’s disease.

Retrospective cell birth dating techniques have enabled scientists to examine DNA synthesis and the generation of new cells and determine the age of cells by assessing temporal changes in the levels of isotope carbon-14 in genomic DNA [84]. This has enabled the quantification of cellular turnover under physiological and pathological conditions. Comprehensive transcriptomic profiling and the western blot analysis of postmortem brain tissues from adults of different ages, sexes and ethnicities have revealed high DCX and PSA-NCAM expression levels in the striatum and the absence of the age pigment lipofuscin in patients with neurological diseases [85], which suggests the presence of newly generated neurons and neuronal precursor cell (neuroblast) formation in these patients [86,87,88,89,90]. These neurons were identified as calretinin (CR)-positive GABAergic interneurons [91]. Furthermore, increased striatal neuron recovery was observed in the ischemic striatum after growth factor treatment, followed by improvements in motor behavior [92,93]. Notably, studies have shown a significant increase in adult striatal neurogenesis in humans compared to rodents [91], suggesting the translational value of evaluating striatal neurogenesis in nonhuman animal models. There is controversy regarding whether newly generated striatal neurons are derived from cells that migrate from the SVZ [94,95] or local progenitors in the striatum [96,97]. Striatal neurogenesis may be regulated by external factors such as disease progression. For example, compared to patients with late-stage Huntington’s disease, patients with early-stage Huntington’s disease show a more modest decrease in postnatal neurogenesis [85], and drugs such as methamphetamine [98] and pramipexole induce some striatal neurogenesis [99]. Although the role of newly generated neurons is unknown, recent results indicate that the manipulation of striatal neurogenesis [100] may be a novel therapeutic approach for neurodegenerative diseases such as Huntington’s disease and Parkinson’s disease as these strategies can induce neurogenesis or, in the case of cerebral ischemia, functional recovery after injury.

#### 2.3.5. Amygdala

The human amygdala comprises 13 nuclei in the rostral temporal lobe that function to assign emotional salience to a vast spectrum of external stimuli and encode memories largely through fear learning and anxiety. Amygdala dysfunction has been implicated in the pathophysiology of human mood and anxiety disorders [58,101]. For the first time, adult-born neurons in the amygdala were observed in monkeys using the thymidine analog BrdU, the dye 1,1′-dioctadecyl-3,3,3′,3′-tetra-methylindo-carbocyanin and the coimmunostaining of various neurogenesis markers such as PSA-NCAM, βIII-Tubulin and collapsing response mediator protein-4 [102,103]. Using this method, it was found that up to 27% of cells within the temporal lobe were newly generated cells, and the authors speculated that these neurons may have originated from the SVZ [102]. Similarly, newborn neurons expressthe Achaete-scute complex homolog 1 (Ascl1) [104] or are double-labeled with DCX and BrdU and have electrophysiological properties seen mainly in interneurons were detected in subregions of the amygdala [103,104,105]. More recently, immature neurons were identified in the amygdala in humans between 4~15 years of age; however, it is unclear whether these neurons can be stimulated to differentiate into mature neurons [106] and whether neurons generated postnatally are supplementary or replace existing neurons. Moreover, the presence of adult NSCs in the amygdala has not been observed. Nevertheless, the delayed maturation of immature neurons regardless of brain region is of immense interest in the fields of neural transplantation, plasticity and repair.

## 3. Extrinsic Factors Associated with Neurogenesis Impairment

Different pathological and physiological stimuli can impact the entire neurogenesis process [107]. Adult hippocampal neurogenesis is influenced by pathological stimuli such as pain and hypoxia. In addition, extrinsic factors such as alcohol, radiation exposure and malnutrition can also regulate neurogenesis [44]. We will describe in detail the effects of these physiologically and pathologically extrinsic factors on neurogenesis (Table 1).

### 3.1. Alcohol and Stress

Different pathological and physiological stimuli can impact the entire neurogenesis process [108]. There is clear evidence that excessive alcohol consumption extensively damages multiple organ systems, especially the CNS, as chronic alcohol use leads to permanent cognitive decline and motor deficits [109]. In vitro and in vivo studies have shown that prenatal alcohol exposure impairs the development of the newly formed cortex in the developing fetus [110,111], and chronic alcohol exposure reduces neurogenesis and enhances cell death in adolescents; these changes may not be alleviated until a substantial period of time after alcohol cessation [112,113]. High-dose alcohol exposure in young adult rats suppresses hippocampal neurogenesis, including by decreasing the number of type-1 NSCs and type-2a NPCs in the DG subgranular zone and FOS+ granule cells, and reduces synaptic plasticity [114]. However, the mechanisms underlying neurogenesis impairment following alcohol consumption exposure remain unclear. At present, there is some evidence that the expression of hippocampal brain-derived neurotrophic factor (BDNF), a factor that regulates the survival and differentiation of newborn neurons, is suppressed and neurogenesis is interrupted by alcohol use during adolescence. Interestingly, neurogenesis impairment and depression-like symptoms induced by repeated alcohol use in adolescence are reversed after BDNF agonist administration during alcohol withdrawal and abstinence [115], indicating that alcohol likely interrupts adult neurogenesis via the suppression of hippocampal BDNF expression. Chronic binge-like alcohol consumption during adolescence also causes persistent neural and behavioral alterations due to the induction of neuroinflammation [116]. For instance, interleukin 1β (IL-1β) expression is increased in lipopolysaccharide (LPS)- and ethanol-treated mice [117] and has been repeatedly identified in other models to inhibit various stages of neurogenesis, such as proliferation and maturation [118]. Additionally, studies have shown that the effects of alcohol exposure in adolescent animals are usually more pronounced than the effects of comparable alcohol exposure in adult animals [119], suggesting that the adolescent brain may be more susceptible to alcohol-induced neurotoxicity. However, the dose of alcohol, the duration and pattern of alcohol exposure and the age of the organism are closely associated with the degree to which each stage of adult neurogenesis is affected [120,121].

Another important extrinsic factor is stress. Stress impairs hippocampal neurogenesis, induces anxiety- and depressive-like behaviors and is known to result in increased drinking [122,123]. Stress can be the result of external factors such as the presence of natural predators or intruders or occupying a low position in the social hierarchy (subordination hierarchies) or by factors that interfere with physiological function such as alcohol consumption. Regardless of its origins, stress dramatically decreases NSC proliferation, and this change persists throughout exposure to chronic stress and is associated with a reduction in the size of the DG and with poor learning performance [124]; in addition, it contributes to the development of alcohol addiction [125]. Stressors, including alcohol, activate the neuroendocrine hypothalamic–pituitary–adrenal (HPA) axis, which regulates the body’s stress responses [126]. The activation of the HPA axis causes the adrenal glands to secrete steroid hormones such as corticosterone in rodents, and this process may underlie the effects of alcohol and other stressors on neurogenesis [127,128]. The relationship between corticosterone and neurogenesis is discussed in more detail below.

### 3.2. Radiation Exposure

Radiation exposure is also an important extrinsic factor that increases the risk for cancers and noncancerous diseases, such as atherosclerosis and neurodegeneration [129]. Brain tumors are usually treated with radiation therapy and concomitant temozolomide chemotherapy. However, radiation treatment is accompanied by side effects that are closely associated with behavioral impairments such as cognitive deficits and anxiety [129,130,131]. Studies have shown that radiation exposure leads to progressive neurogenesis impairment accompanied by a decrease in the number of stem and neuroprogenitor cells, a reduction in hippocampal neuronal dendritic arborization, degenerative changes and the loss of DG neurons, possibly by inducing apoptosis, neuroinflammation and accumulated DNA damage [132,133,134,135]. Therefore, it is possible that progressive learning and memory deficits following irradiation are caused by the impairment of neurogenesis in the hippocampal DG. Furthermore, studies of the effects of brain irradiation in humans and animals have reported that microcephaly, aging, mental retardation, seizures and brain tumors are induced by prenatal radiation exposure [136,137,138,139], likely due to hippocampal dysfunction resulting from the long-term abolishment of normal NSC/NPC activity.

### 3.3. Traumatic Brain Injury and Hypoxia

Traumatic brain injury (TBI) is a leading cause of injury-related mortality and morbidity worldwide and is characterized by cognitive dysfunction and neuroinflammation. Traumatic brain injury symptoms range from mild to severe depending on the site and severity of the injury and volume of damaged tissue. The risk of neurodegenerative diseases, such as AD and PD, is increased in patients with TBI [140,141]. It is known that adult hippocampal neurogenesis plays an important role in cognitive function in rodents [142]. The changes in neurogenesis that occur after TBI have mainly been studied in animal models due to the limitations of human studies. There are no significant changes in neurogenesis following mild TBI, suggesting that the limited cortical cell death induced by mild TBI may not be enough to trigger neurogenesis as a compensatory response. However, moderate TBI promotes NSC proliferation, and severe TBI promotes all three stages of neurogenesis, proliferation, differentiation and maturation, possibly by inducing severe neuronal death [141,143]. Similarly, increased cell proliferation is observed in the acute stage of moderate focal TBI in mice, while net hippocampal neurogenesis remains unchanged, which might be because NSC proliferation is transiently increased and because NSCs mostly differentiate into astrocytes rather than neurons [144]. Moreover, in pediatric patients TBI significantly impairs hippocampus-dependent cognitive functions, decreases the survival rate, causes the ectopic migration of adult-born neurons, increases astrogenesis in the hilus of the DG and induces neuroinflammation [145,146]. The extent to which neurogenesis is affected is associated with the degree of injury, and the degree to which TBI temporarily promotes hippocampal neurogenesis increases as the injury severity increases, which results in the compensatory loss of mature neurons. Furthermore, longitudinal studies show that recovery after mild to moderate TBI is asymptotic in human patients, with rapid recovery in the first weeks and months of injury followed by a slower rate of improvement as well as a plateau or even deterioration afterward [147]. Increasing evidence shows that the BDNF level is increased by drug treatment after TBI and that this increase in BDNF expression promotes neuronal survival and neurogenesis [148,149]. Traumatic brain injury can exacerbate neuroinflammation by regulating microglia, and microglia play a dual role in neurogenesis depending on the cellular microenvironment [150,151]. Thus, it is possible that TBI acutely increases the generation of new neurons by regulating neuroinflammation or BDNF expression and subsequently induces a chronic decrease in baseline neurogenesis, which may be due to the loss of NPCs and/or the death of immature hippocampal neurons [146]. In addition, newly born neurons generated after TBI may also exhibit morphological and functional aberrations that can contribute to pathogenesis. For example, it has been shown that abnormal migration and impaired dendrite development in newly born neurons occurs in a TBI model, and these changes may result in the dysregulation of neural network connectivity [145,152]. Therefore, the effect of TBI on neurogenesis may result in a complex set of beneficial and pathological alterations.

An uninterrupted supply of oxygen (O2) to the brain is necessary to maintain cellular homeostasis and energy metabolism [153]. It has been reported that hypoxia plays an important role in the regulation of multiple biological processes, including neurogenesis, angiogenesis and erythropoiesis, by controlling the expression of related proteins, which facilitates neuronal cell survival in environments with low O2 concentrations [153,154]. Studies have shown that mild hypoxia exerts neuroprotective effects in response to multiple stressors to which the brain develops tolerance, while severe hypoxia can cause neurodegeneration [155,156]. Under mild hypoxia, NSC proliferation and differentiation are promoted in vitro [157], and neurogenesis is also stimulated in the hippocampal DG of adult rats in vivo [158,159]. In line with these findings, other evidence shows that chronic intermittent hypobaric hypoxia (CIHH) alleviates cognitive impairment, increases hippocampal neurogenesis and enhances synaptic plasticity in epileptic rats through the promotion of Wnt/β-catenin pathway activity [160]. This result is further supported by an in vitro study showing that hypoxia stimulates early neurogenesis in human fetal NSCs by activating the Wnt pathway [161]. In addition, hypoxia induces the differentiation of hypoxia-responsive recovering cells into neurons and neuronal apoptosis in the brain of zebrafish embryos [162] and enhances the axonal outgrowth of cultured neurons [163]. However, the mechanism underlying the effect of severe hypoxia on neurogenesis remains unclear. It has been reported that hypobaric hypoxia impairs adult neurogenesis and social interaction through the cyclooxygenase-1/EP1 receptor pathway-mediated activation of the NLRP3 inflammasome [164]. Furthermore, accumulating evidence suggests that hypoxia contributes to the progression of AD by inducing amyloid β peptide (Aβ) aggregation, tau hyperphosphorylation, BBB dysfunction and the impairment of calcium homeostasis [153,165,166], indicating that hypoxia is likely to be closely related to neuronal degeneration. Additionally, hypoxia increases β-secretase 1 (BACE1) gene transcription, which leads to enhanced BACE1 secretase activity and the accumulation of neurotoxic Aβ [167,168]. Previous evidence has shown that Aβ aggregation and tau hyperphosphorylation negatively regulate hippocampal neurogenesis [169,170]. In addition, our previous studies suggested that amyloid precursor protein (APP) inhibits neurogenesis via interaction with the transcription factor FOXO3a or vacuolar protein sorting-associated protein 35 D620N mutant [171,172]. Therefore, we speculate that severe hypoxia negatively modulates neurogenesis and causes neurodegeneration by stimulating Aβ aggregation, tau hyperphosphorylation, BBB dysfunction and neuroinflammation and impairing calcium homeostasis.

### 3.4. Malnutrition

Proper nutrition is important for CNS maturation and functional development. Preclinical studies indicate that high-calorie diet consumption impairs adult neurogenesis, leading to reductions in the proliferation of cells and the differentiation of neuroblasts/neurons and memory deficits in rats and mice [173,174,175]. In contrast, intermittent fasting or caloric restriction has a positive effect on adult neurogenesis [176,177,178]; different types of malnutrition may affect the maturation of the brain and cognitive function, resulting in behavioral abnormalities and disturbances in learning and memory. Previous studies have found that malnutrition can decrease body and brain weight, impair cognitive function and neurotransmission and alter protein phosphorylation and oxidative status in the brain [179,180,181,182]. One study found that malnourished mice exhibit poor spatial memory and learning and decreased plasticity as well as alterations in microglia [183]. It has been shown that the earlier the onset of nutritional deficiency, the more severe and lasting its effects [184]. Numerous studies have investigated the effects of prenatal or perinatal malnutrition on brain development. For example, nutritional restriction during gestation interrupts cell division and decreases the number of cells in all organs even after adequate refeeding, whereas malnutrition at later stages decreases the protein/DNA ratio (cell size), which is rescued after refeeding [185]. Another finding consistent with these results is that maternal malnutrition from conception is sufficient to reduce neurogenesis and cause memory loss in adult offspring [186]. Maternal malnutrition induces persistent deficits in learning in offspring, possibly due to impairments in pattern separation, and is associated with the reduced production of new adult hippocampal neurons. However, the detailed mechanism by which malnutrition affects neurogenesis remains unknown.

### 3.5. SARS-CoV-2

In late 2019, a viral outbreak of a novel zoonotic enveloped RNA betacoronavirus of the family Coronaviridae [187] that is pathogenic to humans, namely, severe acute respiratory syndrome coronavirus 2 (SARS-CoV-2), was first identified in Wuhan, Hubei Province, China. With 79.5% sequence homology to severe acute respiratory syndrome coronavirus 1 (SARS-CoV-1), SARS-CoV-2 infection causes symptoms such as dyspnea, fatigue, pulmonary insufficiency, fever, dry cough, nasal congestion and even death and is known as coronavirus disease 2019 (COVID-19) [188,189,190]. Unfortunately, the high risk of recurrence of COVID-19 after recovery poses an enormous threat to the economy, society and health care systems [191,192].

**Figure 3:**
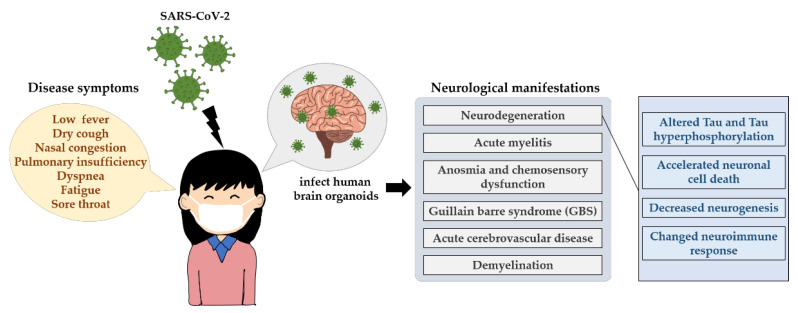
The neurological influences of SARS-CoV-2 in humans. The main transmission routes of SARS-CoV-2 are direct transmission, aerosol transmission and contact transmission, which induces an acute respiratory infectious disease, showing some signature disease symptoms such as low fever, dry cough, nasal congestion and even death. SARS-CoV-2 can infect human brain organoids and can lead to persistent neurological symptoms, for example neurodegeneration. It displays altered Tau, Tau hyperphosphorylation at T231, neuronal cell death or death of nearby cells, neuroimmune response as well as neurogenesis.

Studies of previously known coronaviruses, such as SARS-CoV-1 and Middle East respiratory syndrome-related coronavirus (MERS-CoV), have shown that viral entry relies on the binding of angiotensin-converting enzyme 2 (ACE2) [193] and dipeptidyl peptidase-4 (DPP4) [194], respectively, to host cell receptors. Similarly, in the case of SARS-CoV-2, the ACE2 receptor has been identified as the main receptor for viral entry into host cells [195,196]. Its distribution in brain areas such as the middle temporal gyrus, posterior cingulate cortex, thalamus, substantia nigra and hippocampus [197,198] and in the CSF [199] is highly heterogeneous. Moreover, numerous reports of COVID-19-associated neurological manifestations such as acute myelitis [200], anosmia and chemosensory dysfunction [201], guillain barre syndrome (GBS) [202,203,204,205] and acute cerebrovascular disease [206,207] have been reported. Coronavirus infection can lead to demyelination [208] and neurodegeneration [209,210]. Furthermore, these neurological symptoms seem to persist in some recovered COVID-19 patients [211,212,213,214,215,216,217]. The investigation of the long-term impact of SARS-CoV-2 infection in the brain is essential (Figure 3).

Interestingly, a clinical comparison of microstructural and volumetric changes between recovered COVID-19 patients’ brains and the brains of individuals without COVID-19 revealed that recovered COVID-19 patients show the regional enlargement of brain areas, especially in the central olfactory system [211], which is thought to be the first region of the CNS that SARS-CoV-2 infects [218,219]. In this study, the authors postulated that the enlargement of such brain regions could potentially be due to neurogenesis, hypertrophy and remyelination in white matter pathways [211]. Additionally, the gray matter volume in the hippocampus and cingulate gyri was negatively associated with the loss of smell and loss of memory 3 months after recovery in COVID-19 patients, suggesting a possible role for neurogenesis in these regions. Furthermore, the density of neuronal progenitor cells and newborn neurons was decreased in patients diagnosed with COVID-19 compared with control subjects [220]. However, there was no evidence demonstrating the mechanism by which COVID-19 regulates neurogenesis. Significant neuropathological changes, including hypoxic damage, microglial activation, astrogliosis, leukocytic infiltration and microhemorrhages [221,222], have been observed in COVID-19 patient postmortems. Nonsurvivors of COVID-19 have been found to show hemorrhage and posterior reversible encephalopathy syndrome-related brain lesions, and SARS-CoV-2 infection could be the leading cause of neuronal stress and inflammation [223]. In addition, previous pathological studies demonstrated a COVID-19-induced reduction in the neuronal number in brain regions, with adult hippocampal neurogenesis being decreased [219,224] and the neuroimmune response being increased [221,222,225,226], and similar changes have also been observed in SARS-CoV-2-infected human brain organoids [227]. Notably, prolonged neuroinflammation is known to negatively regulate neurogenesis, although its effect largely depends on the brain microenvironment, such as the type of inflammatory cytokine expressed [228,229,230,231,232]. Interestingly, SARS-CoV-2 was capable of infecting human neural progenitor cells as well as brain organoids [233], with infected organoids displaying alterations in tau, the hyperphosphorylation of tau at T231, neuronal cell death [234] or the death of nearby cells [235]. Overall, inconsistent with the hypothesis derived from anatomical volumetric studies performed by Lu et al., there seem to be more data suggesting that SARS-CoV-2 infection impairs neurogenesis or is associated with accelerated neuronal cell death.

There is a lack of adequate sample size, sex, age and neurologic symptom and stage matching across these studies, and the precise mechanisms of COVID-19-induced neurogenesis and neurological deficits remain largely unknown. Nevertheless, the neuropathological and psychiatric complications of COVID-19 (including in patients who have recovered and asymptomatic patients) need to be further elucidated as they represent major public health challenges.

## 4. Intrinsic Factors Associated with Neurogenesis Impairment

While external factors affect neurogenesis, some intrinsic factors also play an important role in the regulation of neurogenesis. The possible mechanisms involved in the regulation of neurogenesis by these intrinsic factors are described in detail below and summarized in Table 2.

### 4.1. Hormone Imbalances

Hormones, which play an important role in coordinating physiological processes such as metabolism, growth and development, are chemicals synthesized by highly differentiated endocrine cells and secreted directly into the blood. It has been reported that different kinds of hormones, including sex, stress and metabolic hormones, are involved in regulating embryonic and adult neurogenesis and dendritic morphology [236]. Both females and males secrete sex hormones that are roughly classified as estrogens, androgens and progestogens in different concentrations. High endogenous estrogen levels during proestrus and exogenous estrogen treatment appear to increase NSC proliferation and negatively regulate cell death in rats and mice [237,238]. Estradiol is the most abundant and active estrogen in many species and seems to enhance mouse embryonic stem cell proliferation in vitro by increasing store-operated calcium entry and activating the nuclear factor of activated T cells [239]. Estradiol also promotes cell proliferation and inhibits cell death in the SGZ by modulating the activity of estrogen receptors [236]. Studies have shown that treatment with exogenous estradiol inhibits the decrease in NSC neurogenesis, increase in cell death and impairment of cognition induced by exposure to ketamine by modulating the estrogen receptor β pathway associated with GSK-3β [240,241]. In addition, high exogenous and endogenous estradiol levels may increase the dendritic spine density in CA1 hippocampal pyramidal neurons, and dendritic morphology is regulated in a manner dependent on the concentration of estradiol [242,243]. Increasing evidence indicates that estrogens maintain neurogenesis, neuronal activity and synaptic plasticity [244,245].

The most striking difference in brain morphology between males and females is size, with males having, on average, a larger brain than females [246]. Kelava et al. found that the androgens testosterone and dihydrotestosterone increase the proliferation of cortical progenitors and the size of the neurogenic pool in both male and female brain organoids, suggesting that the presence of androgens leads to a primarily cell-extrinsic driven sex difference [247]. Testosterone and its metabolite dihydrotestosterone may also be involved in modulating neurogenesis, but testosterone can also be metabolized to estradiol via the enzyme aromatase. Thus, it is difficult to determine whether testosterone or estradiol plays a role in neurogenesis [236]. However, one study found that cell proliferation in the SVZ was enhanced by testosterone, not dihydrotestosterone, in males, but no changes were seen in females [248]. Both testosterone and dihydrotestosterone increase the spine density in hippocampal CA1 neurons in male rats [249] and play a role in cell survival via an androgen receptor-dependent pathway [250,251]. However, the abuse of anabolic steroids can influence brain function and further cause behavioral changes. One study found that the testosterone analog 19-nortestosterone inhibited the proliferation of cultured embryonic and adult rat NSCs and decreased the number of newborn neurons in the DG of rats, suggesting that testosterone analogs may seriously influence the generation of NSCs and lead to long-term negative effects in the brain [252]. Interestingly, dehydroepiandrosterone can stimulate neurogenesis in P19 mouse embryonic carcinoma cell- and human embryonic stem cell-derived neural progenitors in vitro and induce dopaminergic neuron differentiation, which is caused by an increase in neurogenesis and a decline in apoptosis [253]. In cultured hippocampal progenitor cells from female humans, estrogen or testosterone treatment increased the cell density but did not affect cell proliferation or cell death. In addition, prolactin induces a transient increase in differentiation but a reduction in cell proliferation at the same timepoint [254]. Therefore, sex hormones likely have a short-term impact on neurogenesis in human hippocampal progenitor cell culture. The effects of progesterone levels on neurogenesis remain unclear. Studies have shown that exogenous progesterone treatment has neuroprotective effects as it promotes neurogenesis and improves cognition and neurologic function in vivo after brain injuries, such as severe global cerebral ischemia and ischemic stroke, likely through the regulation of endogenous BDNF levels [255,256].

Once the body perceives a stress, danger, or threat in the environment, it responds by releasing stress hormones that stimulate the energy storage system and activate the sympathetic system [257]. Chronic unpredictable stress elevates corticosterone levels, impairs adult hippocampal neurogenesis and induces depression-like behavior in male animals [258,259]. However, acute exposure to foot shock or psychological stressors does not alter cell proliferation [260,261]. Surprisingly, acute stress decreases hippocampal cell survival [261] and decreases the density of both apical and basal dendrites in the hippocampal CA1 region of female animals [262]. Therefore, there are differences in the effects of chronic and acute stress on the sex hormone-mediated regulation of neurogenesis and spine density. Additionally, thyroid hormones, which are metabolic hormones, have been found to increase the survival of newborn neurons, modulate adult hippocampal neurogenesis and regulate BDNF levels but not regulate cell proliferation [263]. In addition, exogenous thyroid hormone treatment alleviates the methamphetamine-induced impairment of neurogenesis, reduction in mitochondrial biogenesis and memory decline in rats [264]. In general, hormones have marked effects on the modulation of cell proliferation, differentiation and survival and spine density during embryonic development and adulthood.

### 4.2. Gut Microbiome

The majority of colonizing microbes in the human body exist within the gastrointestinal tract [265]. In recent decades, the importance of crosstalk between the gut microbiome and the brain (also known as the microbiota–gut–brain axis) and its involvement in regulating neurogenesis, behavioral functions and neuropsychiatric conditions has become apparent [266,267,268,269,270,271]. The study of the bidirectional interaction between gut bacteria and the brain began with the investigation of the potential beneficial effects of postnatal microbial colonization on brain plasticity. Initial studies focused on the neuroendocrine system, specifically the response of the HPA to restraint stress in germ-free (GF), specific pathogen-free (SPF) and gnotobiotic mice [270,272]. Studies have shown that GF mice show decreased cortical and hippocampal levels of neurotrophic factors, including BDNF, and an increase in plasma ACTH levels and that microbial exposure in the early development stage is critical for the development of a normal HPA-mediated stress response in mice and the regulation of synaptic protein expression [272,273]. Studies using GF mice demonstrated that microbes modulate the expression of genes involved in signaling pathways, synaptic transmission and neurotransmitter turnover in the cortex, striatum, cerebellum, hypothalamus and dorsal hippocampus [274]. Collectively, these regions are implicated in motor control, anxiety-like behavior and memory consolidation [270].

Given the important effects of the gut microbiota early in life [270,274], it is speculated that the gut–brain axis could play an influential role during the dynamic phase of postnatal neurogenesis, thus impacting subsequent brain development and altering cognitive functions and behavior. Early processes involved in brain development include cell differentiation, axonal myelination and synaptogenesis, and these processes contribute to infant cognition. Interestingly, when the gut microbiota of aged donor mice was transplanted into young GF mice, the mice exhibited enhanced hippocampal neurogenesis, but this phenotype was not observed in aged GF mice that received transplantation of the gut microbiota of aged donor mice [275], further suggesting that the response of neurogenesis to the gut microbiota is age-dependent. Surprisingly, when healthy adult mice were treated with antibiotics for 7 weeks, long-term neurogenesis in the SGZ of the DG was significantly impaired, but this change could be fully reversed through probiotic administration and voluntary exercise [276]. Moreover, an interesting study showed that C57BL/6 mice that received fecal microbiota transplantation from 5xFAD mice displayed decreased adult hippocampal neurogenesis and brain-derived neurotrophic factor expression and increased p21 expression, resulting in memory impairment and microglial activation [277]. Gut microbes can also regulate microglia and cognitive function during malnutrition and regulate enteric neurons and glia [183,278]. It has been postulated that bidirectional communication between the intestinal microbiome and the brain occurs through the vagus nerve, which regulates hippocampal plasticity and maintains newly born cells [279]. More recently, a gut microbe-derived metabolite, indole, was identified to be involved in the regulation of adult hippocampal neurogenesis and neuronal maturation in mice and, for the first time, aryl hydrocarbon receptor (AhR) signaling was found to regulate neurogenesis within the microbiota–gut–brain axis [280].

This information highlights the potential of novel therapeutic strategies targeting the gut–brain axis to either slow cognitive decline or promote neuronal regeneration or neuronal repair mechanisms. Unfortunately, most of these studies were performed in animals of varying ages, and the mechanisms underlying intestinal microbiome-induced neurogenesis and the effects of intestinal microbes on brain functions are unclear due to the complicated orchestration of a wide variety of gut microbes and immune cells. In addition, it is difficult to know if the findings are translatable to human subjects. Further studies are required to better delineate the regulatory mechanism of bidirectional gut–brain communication in adult neurogenesis and cognitive function.

### 4.3. Aging and Alterations to Neuroimmunity

Aging is an unavoidable nonpathological process that can link health and disease, and it is one of the most important risk factors for compromised brain function and plasticity, including the disruption of adult hippocampal neurogenesis-mediated structural/functional plasticity and mood modulation. Although neurogenesis occurs throughout life, a marked reduction in adult hippocampal neurogenesis along with aging was observed in the hippocampal DG of rodents, carnivores, nonhuman primates and humans, showing that the number of RGLs and intermediate progenitors, the proliferation and differentiation of NSCs/NPCs and the survival rate of adult newborn neurons are decreased [87,281,282,283,284,285]. These findings are presumably influenced by cell-intrinsic and cell-extrinsic alterations with age. Adult neurogenesis is strictly regulated through environmental conditions affecting the NSC pool, which is composed of numerous cell types, such as microglia and astrocytes. It has been demonstrated that a large number of NSCs/NPCs differentiate into glial cells but not neurons during aging [87]. Intriguingly, in parallel with age, astrocytes are generated by the differentiation of activated RGLS after several rounds of cell division, which leads to a reduction in the adult neurogenic niche and further decreases adult hippocampal neurogenesis through the uptake or exocytosis of diverse molecules (for example, soluble factors such as lactate and triphosphate) [286,287]. In addition, microglia can regulate the development of adult newborn neurons by releasing trophic factors (such as BDNF and IGF1) and cytokines (such as tumor necrosis factor (TNF)-α) [288,289] and have phagocytic capacity and crosstalk with neurons through fractalkine-CX3CR1 signaling to maintain the environment of the NSC pool [290].

In addition to intrinsic cellular alterations, changes in the systemic environment during aging are important factors influencing adult neurogenesis. Using a model of heterochronic parabiosis, it has been demonstrated that immune-linked molecular and cellular changes in the systemic environment that occur with aging inhibit adult neurogenesis [291,292]. In these studies, the exposure of young heterochronic parabionts to old blood resulted in decreased proliferation and differentiation in vivo and in vitro, while neurogenesis in aged heterochronic parabionts exposed to young blood was increased [291,292]. Subsequently, utilizing a proteomics approach, Wyss-Coray and colleagues found that the levels of several factors, such as CCL11, CCL12 and B2M, are increased in the blood of healthy aged people and are correlated with the age-linked reduction in adult neurogenesis [291,293,294,295]. Interestingly, the stereotaxic injection of B2M and CCL11 into the DG and the administration of B2M and CCL11 in vitro can also negatively regulate neurogenesis [291,293]. However, the underlying cellular source leading to the increase in the systematic levels of age-related factors in the blood of aged patients remains unclear. Based on the immune origin of these factors, the proinflammatory remodeling of the peripheral immune system may account for this change. In addition, the age-related pro-aging factors that prevent the stimulation of adult neurogenesis in the aged brain remain unknown. To date, there is no good way to slow aging, and thus the enhancement of neurogenesis is beneficial during aging.

## 5. Future Prospective

**Figure 4:**
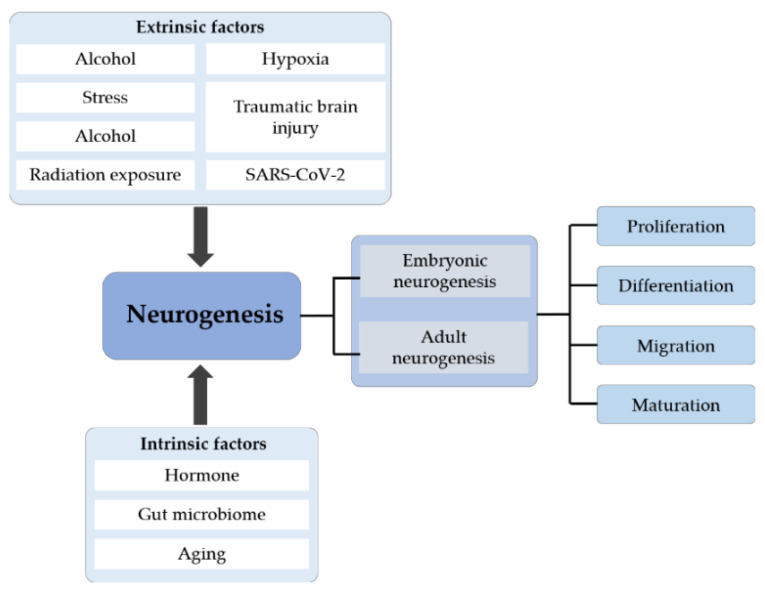
The systematic summary diagram of neurogenesis. Neurogenesis is divided into embryonic and adult neurogenesis that includes four stages: proliferation, differentiation, migration and maturation. Different extrinsic and intrinsic factors can affect the whole process of neurogenesis.

Neurogenesis, especially adult hippocampal neurogenesis, which plays an important role in several hippocampus-dependent functions, is now a key topic in neuroscience. A large number of studies have elucidated the process by which cells are initially formed and identified when and where these cells undergo cell division and then differentiate. The process of differentiation is complex and long, and it includes various steps, such as migration, morphological and molecular differentiation and the final integration of new neurons into preexisting neural networks (Figure 4). As elucidated above, both extrinsic and intrinsic factors affect neurogenesis either in a positive or negative manner. Understanding these pathways may provide ways to improve neurogenesis and promote repair, such as through environmental enrichment, odor exposure and aerobic exercise. In addition, recent research in human brain organoids has paved the way for alternative methods to study neurogenesis to aid the comprehensive understanding of the neuropathology of various neurological diseases. More studies are required for the reliable replication and establishment of disease models in the future, which would open doors for novel drug-screening studies and big data analysis for the discovery of therapeutic targets to mitigate disease progression. The development of new effective strategies for the treatment of neurogenesis-associated neurological diseases is of the utmost importance.

## Figures and Tables

**Table 1:** The effects of extrinsic factors on neurogenesis.

Extrinsic Factors	Results	Period; Model	Reference
Alcohol	Disrupts newborn mouse cortical development and decreased neurogenesis	Gestational alcohol consumption	Sambo et al., 2022
	Reduces neurogenesis and induces loss of neurons	Adolescence and adulthood Wistar rats	Macht et al., 2023
	Suppresses hippocampal neurogenesis including decreasing neural stem cells, immediate nascent neural progenitor and granule cells and reduces synaptic plasticity of newborn granule cells	Adult male SD rat of high-dose ethanol exposure	Takahashi et al., 2022
Stress	Impairs hippocampal neurogenesis and induces anxiety and depressive-like behaviors	Adult male Wistar rats with chronic restraint stress	Adachi et al., 2021
	Decreases NSC proliferation	Adult male Wistar Han rats under unpredictable chronic mild stress	Alves et al., 2018
Radiation exposure	Leads to neurogenesis impairment and neuroinflammation and reduces the dendritic arborization of hippocampal neurons under low dose of radiation	Juvenile and adult C57BL/6NCrl mice	Schmal and Rübe, 2022
	Impairs neurogenesis and causes neuronal DNA damage after radiofrequency electromagnetic radiation exposure	Young Wistar rats	Singh et al., 2023
Traumatic brain injury	Promotes NSC proliferation with moderate TBI and severe TBI increases neurogenesis	Adult male C57BL/6 mice	Amanollah et al., 2023; Wang et al., 2016
	Leads to an acute enhancement of newly generated neurons, decreased cognitive functions, decreased survival rates, ectopic migration of adult-born neurons, increased astrogenesis in the hilus of the DG as well as neuroinflammation	Pediatric New Zealand white rabbits	Rizk et al., 2021; Zhang et al., 2020
Hypoxia	Mild hypoxia may display neuroprotective effects and severe hypoxia can cause neurodegeneration	Adult rats	Costa et al., 2021
	Stimulates early neurogenesis in human fetal NSCs by activating the Wnt Pathway Induces hypoxia-responsive recovering cells differentiated into neurons and neuronal apoptosis in the brain of zebrafish embryos Enhances axonal outgrowth of neuronal cultures	Human fetal NSCs Zebrafish embryos Primary embryonic hippocampal neurons	Dey et al., 2023 Zeng et al., 2022 Turlova et al., 2022
Malnutrition	Displays poor spatial memory and learning plasticity, as well as altered microglia Leads to neurogenesis reduction and adult memory loss in offspring	Young female C57BL/6J mice Preimplantation period in mice	Bauer et al., 2022 Gould et al., 2018
	Enhances NSC proliferation in the hippocampal SGZ and improves cognition after TBI by intermittent fasting	Adult C57 BL/6 N mice	Cao et al., 2022
SARS-CoV-2	Induces a decrease in hippocampal neurogenesis and an upregulated activity of neuroimmune response	COVID-19 patients	Stępień et al., 2023 Cosentino et al., 2021

**Table 2:** The effects of intrinsic factors on neurogenesis.

Intrinsic Factors	Results	Period; Model	Reference
Hormone	Increases NSC proliferation and negatively regulates cell death under highly endogenous estrogen levels	Proestrus rats and mice	Felipe, A. et al., 2021; Tibrewal et al., 2018
	Increases proliferation of cortical progenitors and neurogenic pool by androgens	Brain organoids	Kelava et al., 2022
	Promotes neurogenesis and improves cognition and neurologic function in vivo after brain trauma under exogenous progesterone treatment Alleviates the impairments of neurogenesis, mitochondrial biogenesis and memory performance induced by methamphetamine with thyroid hormone treatment	Adult male rats Adult male Wistar rats	Montes et al., 2019 Tamijani et al., 2019
Gut microbiome	Shapes microglia and cognitive function during malnutrition 5xFAD mice fecal microbiota induces decreased adult hippocampal neurogenesis and brain-derived neurotrophic factor expression in normal C57BL/6 mice	Young female C57BL/6J mice Adult 5xFAD andC57BL/6 mice	Bauer et al., 2022 Kim et al., 2021
Aging	Reduces adult hippocampal neurogenesis	mammal species	Chen et al., 2023

## Data Availability

No new data were created or analyzed in this study.
